# A Novel Pathway of Platelet Activation in ACS Mediated by LL-37 Immunoglobulin G Autoantibody Immune Complexes

**DOI:** 10.1016/j.jacbts.2024.04.012

**Published:** 2024-07-22

**Authors:** Paul C. Dimayuga, Kuang-Yuh Chyu, Xiaoning Zhao, Jianchang Zhou, Nicole Wai Man Lio, Fernando Chernomordik, Daniel Berman, Prediman K. Shah, Bojan Cercek

**Affiliations:** a Oppenheimer Atherosclerosis Research Center, Department of Cardiology, Smidt Heart Institute, Cedars-Sinai Medical Center, Los Angeles, California, USA; b Departments of Imaging and Medicine and Burns and Allen Research Institute, Cedars-Sinai Medical Center, Los Angeles, California, USA

**Keywords:** cathelicidin, immune complex, immunothrombosis, platelet

## Abstract

The cathelicidin antimicrobial peptide LL-37 is a self-antigen in neutrophil extracellular traps that provokes autoantibody responses in autoimmune/autoinflammatory conditions. LL-37 immunoglobulin (Ig) G autoantibody levels were measured in subjects with and without atherosclerotic cardiovascular disease assessed using the coronary artery calcium score, in patients who had a future myocardial infarction and in a cohort of acute coronary syndrome (ACS) patients. LL-37 IgG levels were not associated with coronary artery calcium score, but future myocardial infarction patients had significantly higher LL-37 IgG at baseline. Reduced LL-37 IgG in ACS was associated with increased LL-37 IgG–immune complex. ACS plasma increased activated CD62P+ platelets from healthy donors mediated in part by LL-37 IgG–immune complexes and platelet Fc gamma receptor 2a.

## Highlights

• Neutrophil extracellular traps contain LL-37 antimicrobial peptide.
• High levels of LL-37 IgG may predispose atherosclerotic cardiovascular disease patients to acute myocardial infarction.
• Immune complexes formed between LL-37 and LL-37 IgG bind to platelet Fc gamma receptor 2a and activate platelets propagating thrombus formation on ruptured or eroded plaques.

## Visual Abstract

**Figure:**
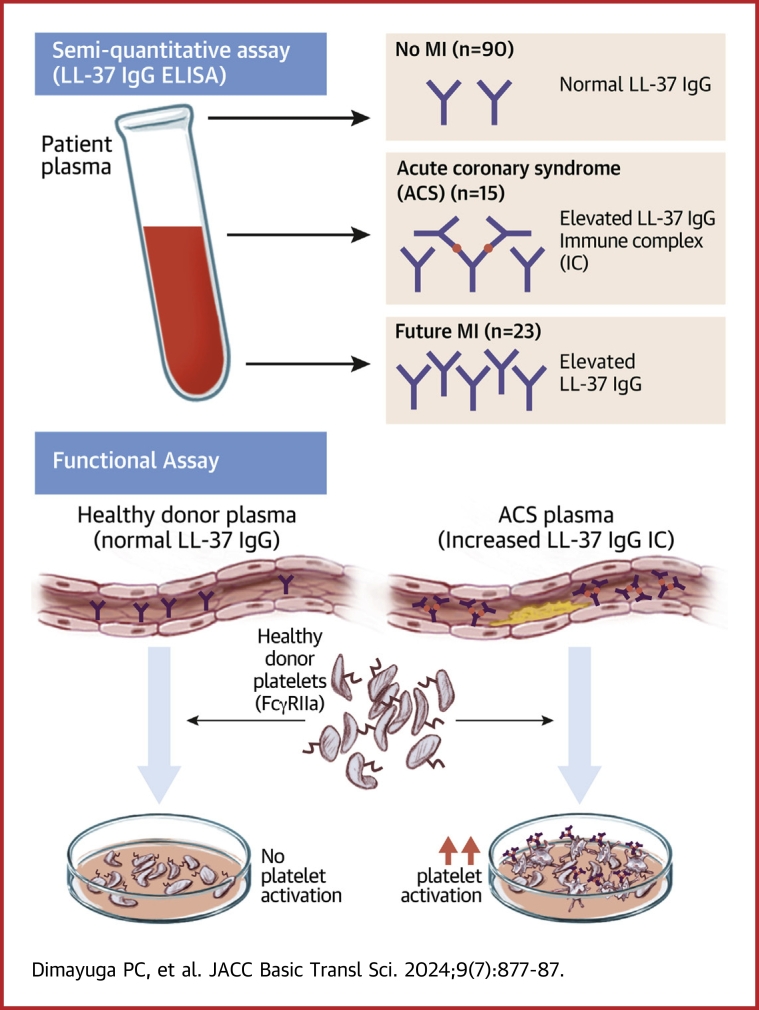

Immunothrombosis is the interlinked function of innate and adaptive immunity with activation of platelets and the coagulation cascade. It is an aspect of host homeostasis that limits pathogen spread and attempts to localize tissue damage.^1^ Unabated inflammation is associated with excessive activation of the immunothrombotic response.^2^ There is growing consideration that unresolved inflammation is linked to the propagation and prolongation of thrombotic responses such as the one in acute coronary syndrome (ACS).^3^^,^^4^

LL-37 is the only known cathelicidin antimicrobial peptide in humans and it promotes inflammatory signaling through several mechanisms.^5^ LL-37 is a component of neutrophil extracellular traps (NETs) that are expelled by degranulating activated neutrophils as part of the innate immune response.^6^ NETs have been implicated in thrombus formation on culprit plaques in ST-segment elevation myocardial infarction (STEMI).^7^ Differential gene expression analysis of a cohort of patients with early-onset STEMI under treatment regimen identified increased expression of LL-37 in peripheral blood mononuclear cells (PBMCs),^8^ and LL-37 is associated with atherosclerotic cardiovascular disease (ASCVD) risk factors.^9^ Systemic levels of LL-37 are reduced in STEMI patients, even as levels in coronary circulation are increased.^10^ In addition, a study showed lower systemic LL-37 levels were associated with a higher risk of a recurrent event.^11^

We previously reported the impairment of T-cell tolerance response to the self-antigen cathelicidin antimicrobial peptide LL-37 in ACS patients.^12^ The impaired tolerance is characterized specifically by increased CD8+CD69+CD137+ T cells. CD137 mediates the generation of immune memory to antigenic challenges.^13^ A potential consequence of T-cell responses to LL-37 is the generation of autoantibodies but it is unknown if this occurs in ACS. Furthermore, the role of autoantibodies to LL-37 in ACS remains unknown.

Pathology attributed to LL-37 in autoimmune diseases includes a feed-forward pathway of inflammatory signaling wherein pre-existing LL-37 autoantibodies form immune complexes (ICs) with LL-37 discharged by activated neutrophils as components of NETs.^6^^,^^14^ These LL-37 immunoglobulin G–immune complexes (LL-37 IgG-ICs) then bind Fc gamma receptor 2a (FcγRIIa) expressed by platelets and neutrophils, which then promotes and amplifies further inflammatory responses.^15^ As such, increased LL-37 IgG levels have been demonstrated in flare-ups of autoimmune diseases such as systemic lupus erythematosus, psoriasis, and rheumatoid arthritis (RA).^16^, ^17^, ^18^, ^19^, ^20^ LL-37 IgG levels in ACS have yet to be investigated.

In this report, we evaluated LL-37 IgG levels in the plasma of ASCVD patients in the context of coronary disease severity assessed using the coronary artery calcium score (CACS). Atherosclerotic plaque rupture or erosion are the main causes of ACS and the CACS is a marker of global coronary atherosclerotic burden and a validated risk prediction tool.^21^^,^^22^ The plasma levels of LL-37 IgG in a subcohort of patients in the EISNER (Early Identification of Subclinical Atherosclerosis by Noninvasive Imaging Research) study^23^ who had coronary calcium scanning, including the subgroup who had a subsequent myocardial infarction (MI), were evaluated. Additionally, the LL-37 IgG levels in a group of ACS patients was also assessed.

The results of the study suggest that subjects with increased LL-37 IgG have a propensity to develop MI in the future and that LL-37 IgG-ICs may be involved in the pathophysiology of ACS by promoting platelet activation.

## Methods

### Patient plasma samples

Residual plasma samples collected at baseline from 113 subjects in the EISNER (Early Identification of Subclinical Atherosclerosis by Noninvasive Imaging Research Trial; NCT00927693)^23^ were evaluated. The EISNER Trial was a community-based cohort of asymptomatic subjects who underwent CAC scanning and cardiovascular risk assessment. Participants were middle-aged with cardiovascular risk factors but no known prior coronary artery disease. Exclusions were any cardiac or cerebrovascular disease or chest pain, age ≥80 years, pregnancy, significant medical comorbidity, or prior coronary calcium scanning. Patient records were reviewed for long-term follow-up (mean: 13.2 ± 2.1 years) to identify patients who experienced MI after scanning. The trial was conducted under Cedars-Sinai Medical Center institutional review board guidelines. Subjects were grouped as No MI or Future MI and characteristics are shown in Table 1. Samples were further classified as CACS <100 or CACS ≥100^24^ and characteristics are shown in Table 2.

**Table 1:** No MI and Future MI Patient Characteristics

	No MI (N = 90)	Future MI (N = 23)	*P* Value
Age, y	59.1 ± 9.8	61.4 ± 9.7	0.42
Female	41 (45.6)	4 (17.4)	0.014
Male	49 (54.4)	19 (82.6)	0.014
BMI, kg/m^2^	28.1 ± 5.4	28.9 ± 3.8	0.42
Cholesterol, mg/dL	208.3 ± 39.8	235.0 ± 26.1	0.003
LDL, mg/dL	128 ± 38.5	160.7 ± 25.2	<0.001
HDL, mg/dL	52.4 ± 17.5	45.3 ± 11.4	0.079
Triglycerides	142.8 ± 83.8	145.7 ± 62.1	0.35
SBP, mm Hg	135.9 ± 17.8	140.2 ± 23.2	0.34
DBP, mm Hg	82.9 ± 11.4	87.3 ± 12.9	0.11
Diabetes	8 (8.9)	0 (0)	0.14
Smoking	11 (12.2)	1 (4.3)	0.27
ACE inhibitor	17 (18.9)	0 (0)	0.024
Beta-blocker	6 (6.7)	3 (13.0)	0.31
Calcium-channel blocker	7 (7.8)	1 (4.3)	0.57
ARB	6 (6.7)	3 (13.0)	0.31
Diuretic	15 (16.7)	1 (4.3)	0.13
Platelet inhibitor	1 (1.1)	0 (0)	0.61
Statin	21 (23.3)	5 (21.7)	0.87
Coronary calcium score	262.7 ± 718.8	482.1 ± 750.0	0.037

Values are as mean ± SD, or n (%) and statistical testing with chi-square test and statistical testing with *t* test or Mann-Whitney test.

ACE = angiotensin-converting enzyme; ARB = angiotensin receptor block; BMI = body mass index; DBP = diastolic blood pressure; HDL = high-density lipoprotein; LDL = low-density lipoprotein; MI = myocardial infarction; SBP = systolic blood pressure.

**Table 2:** No MI and Future MI Patient Characteristics Subgrouped Using CACS

	No MI		Future MI	
	CACS 0-<100 (N = 54)	CACS ≥100 (N = 36)	CACS 0-<100 (N = 9)	CACS ≥100 (N = 13)
Age, y	55.5 ± 9.6	64.3 ± 7.5	56.9 ± 10.3	63.8 ± 8.6
Female	26 (48.1)	15 (41.7)	1 (11.1)	3 (23.1)
Male	28 (51.9)	21 (58.3)	8 (88.9)	10 (76.9)
BMI, kg/m^2^	27.8 ± 5.7	28.5 ± 5.1	29.4 ± 5.1	28.4 ± 3.1
Cholesterol, mg/dL	214.0 ± 40.7	199.9 ± 37.2	241.0 ± 26.7	230.0 ± 26.6
LDL, mg/dL	133.8 ± 39.4	119.5 ± 36.0	166.8 ± 26.5	157.8 ± 25.1
HDL, mg/dL	52.5 ± 17.7	52.1 ± 17.4	43.4 ± 10.5	46.8 ± 12.6
Triglycerides, mg/dL	140.4 ± 80.4	146.6 ± 89.7	154.1 ± 66.8	127 ± 36.3
SBP, mm Hg	131.9 ± 16.2	142.8 ± 18.4	149 ± 28.2	136.4 ± 17.5
DBP, mm Hg	82.3 ± 11.4	83.8 ± 11.7	92.2 ± 15.4	84.5 ± 10.7
Diabetes	6 (11.1)	2 (5.6)	0 (0)	0 (0)
Smoking	7 (13.0)	4 (11.1)	0 (0)	1 (7.7)
ACE inhibitor	8 (14.8)	9 (25)	0 (0)	0 (0)
Beta-blocker	4 (7.4)	2 (5.6)	0 (0)	3 (23.1)
Calcium-channel blocker	5 (9.3)	2 (5.6)	0 (0)	1 (7.7)
ARB	4 (7.4)	2 (5.6)	1 (11.1)	1 (7.7)
Diuretic	6 (11.1)	9 (25)	0 (0)	1 (7.7)
Platelet inhibitor	0 (0)	1 (2.8)	0 (0)	0 (0)
Statin	13 (24.1)	8 (22.2)	0 (0)	5 (38.5)
Coronary calcium score^a^	14.5 ± 24.4	635.0 ± 1037.0	22.4 ± 25.8	800.3 ± 846.0

Values are as mean ± SD, or n (%).

CACS = coronary artery calcium score; other abbreviations as in Table 1.

a 1 future MI patient did not have CACS on record.

A second cohort evaluated was ACS patients who had plasma collected within 72 h of admission to the Cardiac Intensive Care Unit. Patients gave consent under the approved institutional review board protocol Pro00058160. Exclusions were inability to give informed consent, age younger than 18 years of age, active cancer treated with chemotherapy or radiation, patients taking immune-suppressive drugs, and pregnant women. ACS patient characteristics are in shown Table 3. All subjects in the studies gave written informed consent for use of nonidentifiable data.

**Table 3:** ACS Patient Characteristics (N = 15)

Age, y	61.0 ± 10.1
Female	5 (33)
Male	10 (67)
Diabetes	3 (20)
Hypertension	8 (53)
Dyslipidemia	9 (60)
Past or current smoker	3 (20)
Statin use	6 (40)
PCI	15 (100)
1 vessel	10 (67)
2 vessels	2 (13)
3 vessels	3 (20)
WBC	10.3 ± 3.2
Neutrophils, %	62.7 ± 17.0
Lymphocytes, %	26.6 ± 13.9
Neutrophil/lymphocyte	3.7 ± 3.1
Hb	14.3 ± 2.6
Platelet	267.5 ± 53.7
Creatinine	1.0 ± 0.2
BUN	16.5 ± 4.2
Total cholesterol (mg/dL)	181.6 ± 59.8
LDL	114.9 ± 53.4
HDL	45.4 ± 11.1
Troponin first	1.0 ± 2.4
Troponin peak	74.8 ± 68.3
Troponin last	43.0 ± 47.3
LVEF % (echo)	50.7 ± 13.1
STEMI/NSTEMI	13/2
ADHF (%)	2 (13)
CVA	0 (0)

Values are mean ± SD, or n (%).

ADHF = acute decompensated heart failure; BUN = blood urea nitrogen; chol = cholesterol; CVA = cerebrovascular accident; Hb = hemoglobin; LVEF = left ventricular ejection fraction; (N)STEMI = (non) ST-segment elevation myocardial infarction; PCI = percutaneous coronary intervention; STEMI = ST segment myocardial infarction; WBC = white blood cell.

### Anti–LL-37 IgG enzyme-linked immunosorbent assay

Flat-bottomed 96-well polystyrene plates (MaxiSorp) were precoated with 100 μL LL-37 (20 μg/mL) in Na_2_CO_3_-NaHCO_3_ buffer (pH 9.6) overnight at 4°C to assess antibody levels using standard protocol. The coating concentration and serum dilution was optimized in pilot experiments. Horseradish peroxidase anti-human IgG (Southern Biotech) was used to detect antibodies and color development with 2,2′-azino-di-(3-ethylbenzthiazoline sulfonic acid) (Southern Biotech) as a substrate. Optical density values were recorded at 405 nm. Pooled blood type A and B antigen (AB) plasma (Innovative Research) was used as a calibrator for the assay and the values expressed as the ratio of each sample to the calibrator.

### LL-37 IgG-IC enzyme-linked immunosorbent assay

Flat-bottomed 96-well polystyrene plates (MaxiSorp) were precoated with 100 μL sheep anti–LL-37 IgG (1 μg/mL in 1x phosphate-buffered saline [PBS]; R&D Systems) overnight at 4^o^C. Plates were washed and blocked with 2% bovine serum albumin in 1x PBS for 1 h at 37^o^C. Plates were washed and ACS plasma samples diluted 1:100 in 1x PBS were added and incubated for 1 h in 37^o^C. Plates were washed and horseradish peroxidase anti-human IgG (Southern Biotech) were used to detect antibodies with 2,2′-azino-di-(3-ethylbenzthiazoline sulfonic acid) (Southern Biotech) as substrate color development. Optical density values were recorded at 405 nm. AB plasma pooled from healthy subjects (Innovative Research) was used as calibrator for the assay and the values expressed as the ratio of each sample to the calibrator.

### C-reactive protein

C-reactive protein (CRP) levels in EISNER study plasma samples were derived from the existing EISNER database measured using a Luminex Sandwich assay (Alere).^24^ ACS plasma was measured with CRP Quantikine enzyme-linked immunosorbent assay (R&D Systems) as recommended by the manufacturer.

### Healthy donor platelets

Remnant blood samples contained in leukocyte reduction system cones from platelet donors were acquired from the Cedars-Sinai Medical Center blood bank and subjected to PBMC isolation using a modified Ficoll technique as described.^25^ Briefly, blood collected in the leukocyte reduction system cones was reconstituted with an equal portion of PBS containing 2% pooled AB human serum and layered over SepMate Ficoll tubes (Stemcell Technologies). After centrifugation, the layer containing PBMCs and platelets was diluted into 50 mL PBS/2% pooled AB human serum and centrifuged at 350*g* for 10 min without brakes. The supernatant containing the platelets was collected and further centrifuged at 700*g* for 10 min without brakes to pellet the platelets. The platelet pellets were resuspended in 1 mL 2-[4-(2-hydroxyethyl)piperazin-1-yl]ethanesulfonic acid buffer, counted, and used immediately for experiments.

### ACS plasma activation of healthy donor platelets

Four ACS plasma samples that had the highest levels of LL-37 IgG-ICs were selected for platelet activation experiments. Individual plasma samples were diluted to 10% in 2-[4-(2-hydroxyethyl)piperazin-1-yl]ethanesulfonic acid buffer and a fluorescent antibody mix consisting of CD41, CD61, CD62P, and CD154 was added. Platelets isolated from a single donor were then incubated with individual plasma from the ACS patients in duplicates, with the final plasma concentration at 5%. AB plasma pooled from healthy donors incubated with the platelets also at a final concentration of 5% was used as control. The platelets were incubated in the dark at room temperature for 30 min^15^ then 4% paraformaldehyde was added to a final concentration of 1%. The platelets were then pelleted by a 10-min centrifugation at 700*g*, resuspended in fluorescence-activated cell sorting buffer, and analyzed using flow cytometry. Data are presented as percentage of CD41+CD61+ gated platelets The experiment was repeated with 3 different healthy donor platelets.

### Immune-depletion of LL-37 IgG-ICs in ACS plasma

Sheep anti–LL-37 IgG antibody was conjugated to magnetic beads using the Dynabeads Antibody Coupling Kit (Thermo Fisher) according to manufacturer’s protocol. Sheep IgG conjugated to magnetic beads was used as control. Anti–LL-37 beads or IgG beads were then used to immune-deplete the 4 ACS plasma samples with the highest levels of LL-37 IgG-ICs overnight at 4^o^C. Samples were then subjected to magnetic bead separation and the supernatant collected for platelet activation assays as described already in this article. Each experiment included no magnetic bead incubation corresponding to each plasma sample used. Results are expressed as fold-change relative to no magnetic bead incubation. The experiment was repeated with 2 different healthy donor platelets.

### Blocking of FcɣRIIa in platelets

Mouse anti-human FcɣRIIa (clone IV.3, Bioxcell) was preincubated (10 μg/mL) with healthy donor platelets for 15 min at room temperature before addition to individual plasma from the 4 ACS patients with the highest LL-37 IgG-ICs for a final plasma concentration of 5% as described already in this article. Control was no antibody blocking. Plasma samples were premixed with fluorescent antibodies to CD41, CD61, CD62P, and CD154. FcɣRIIa-blocked or control platelets were then added to the plasma samples and incubated in the dark at room temperature for 30 min^15^ then 4% paraformaldehyde was added to a final concentration of 1%. The platelets were then pelleted by a 10-min centrifugation at 700*g*, resuspended in fluorescence-activated cell sorting buffer, and analyzed using flow cytometry. The experiment was repeated with 3 different healthy donor platelets.

### Statistics

Continuous data are presented as mean ± SD or displayed as box and whisker plots (median, maximum, and minimum values). Three or more groups were compared using 1-way analysis of variance with Holm-Sidak post-hoc test for multiple pairwise comparison if normally distributed. Otherwise, Kruskal-Wallis and Dunn post-hoc test was used. Two groups were compared using unpaired or paired *t* test for normally distributed data; otherwise, Mann-Whitney *U* test or Wilcoxon signed rank test was used. Categorical data are presented as count (percentage) and compared using chi-squared test. *P* value <0.05 was considered significant. D’Agostino & Pearson test was used to determine if data were normally distributed. Statistical analysis was performed using GraphPad Prism 10.0.0.

## Results

### LL-37 IgG is elevated in patients with future MI

**Figure 1:**
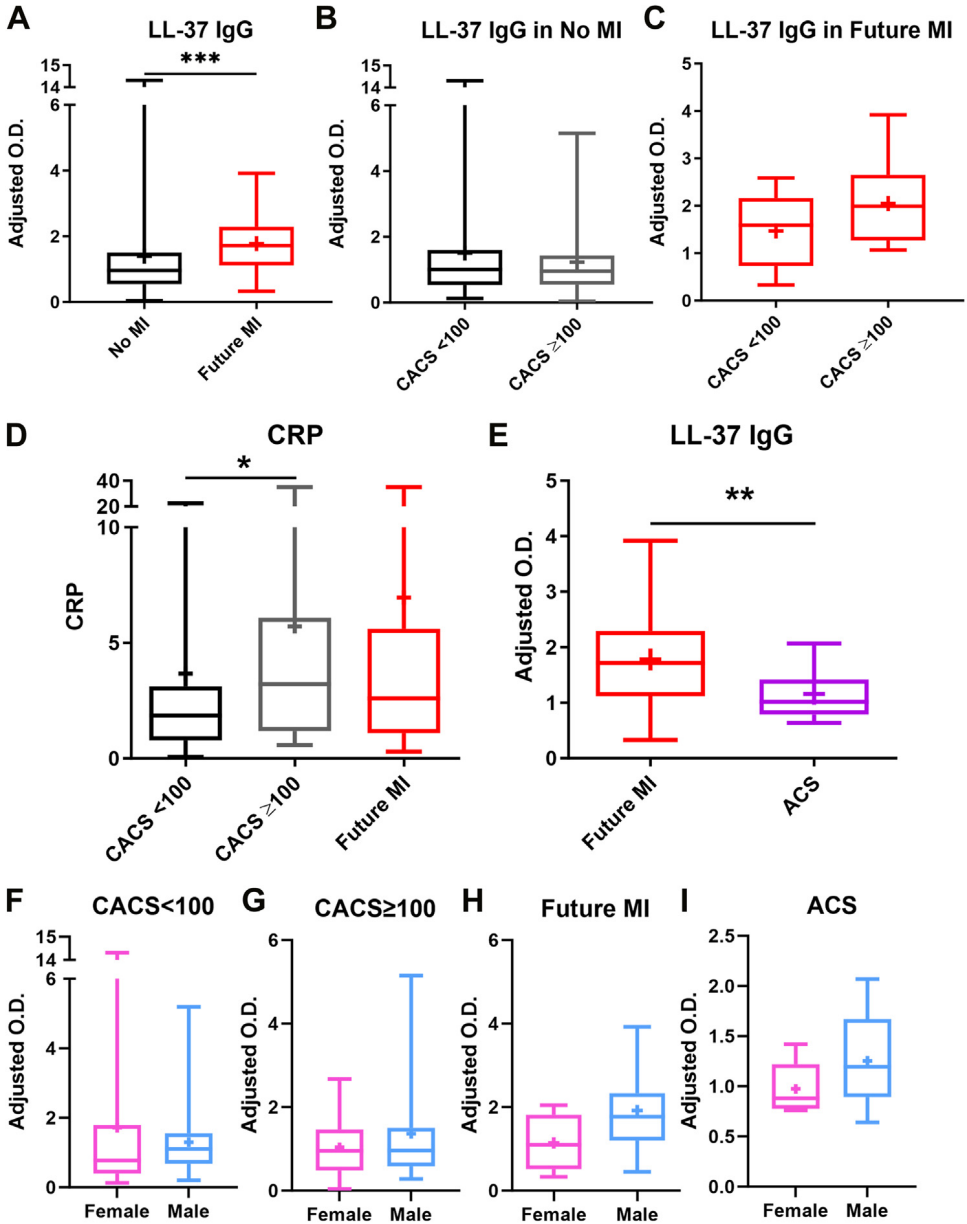
LL-37 IgG in Plasma of ASCVD Patients (A) LL-37 IgG levels in No MI patients (N = 90; black) or those who had Future MI (N = 23; red). (B) No MI patients were further compared based on CACS <100 N = 54 (black); CACS ≥100 N = 36 (grey). (C) Future MI LL-37 IgG levels stratified by CACS <100 (N = 9) or CACS ≥100 (N = 13); 1 patient had no CACS on record. (D) CRP levels in the patients grouped into CACS of <100, ≥100, or those who had Future MI. (E) LL-37 IgG in Future MI patients compared with ACS patients (purple, N = 15). (F-I) LL-37 IgG-ICs compared by sex among the different patient groups (pink = female; blue = male). ∗*P* < 0.05; ∗∗*P* < 0.01; ∗∗∗*P* < 0.001. (A) Mann-Whitney *U* test; (D) Kruskal-Wallis test with Dunn multiple comparison; (E) unpaired *t* test with Welch correction for variance. ACS = acute coronary syndrome; ASCVD = atherosclerotic cardiovascular disease; CACS = coronary artery calcium score; CRP = C-reactive protein; ICs = immune complexes; Ig = immunoglobulin; MI = myocardial infarction.

The relative levels of LL-37 IgG in plasma samples of a subcohort of subjects from the EISNER study^23^ were determined. Characteristics of study subjects as grouped are depicted in Table 1. LL-37 IgG levels were compared between patients who had no MI and those who had a future MI. Patients who had a future MI had significantly higher LL-37 IgG levels compared with those who had no MI (Figure 1A). LL-37 IgG levels grouped according to low risk (CACS 0-<100) or high risk (CACS ≥100)^24^ were not different in No MI (Figure 1B) or Future MI (Figure 1C) groups. The results suggest that LL-37 IgG does not segregate according to CACS. To evaluate the inflammatory status of the patients, CRP levels were compared among the low- and high-risk CACS, and Future MI. CRP levels were significantly higher in CACS ≥100 compared with CACS 0-<100 (Figure 1D). Future MI CRP levels were also elevated but did not reach statistical significance. The results suggested that LL-37 IgG may be associated less with the chronic inflammatory state and more with the acute event. We then evaluated LL-37 IgG in ACS patients and compared it with Future MI patients. There was significantly less LL-37 IgG in ACS patients compared with Future MI (Figure 1E). No significant differences between female and male LL-37 IgG levels were noted among the different patient groups (Figure 1F to 1I) but the low number of females particularly in the Future MI group limits interpretation of this finding. We then considered the possibilities that either LL-37 IgG levels normalized in the acute patients or LL-37 were bound as LL-37 IgG-ICs given the reported increase in LL-37 levels in coronary circulation in MI patients.^10^

### LL-37 IgG-ICs are increased in ACS

**Figure 2:**
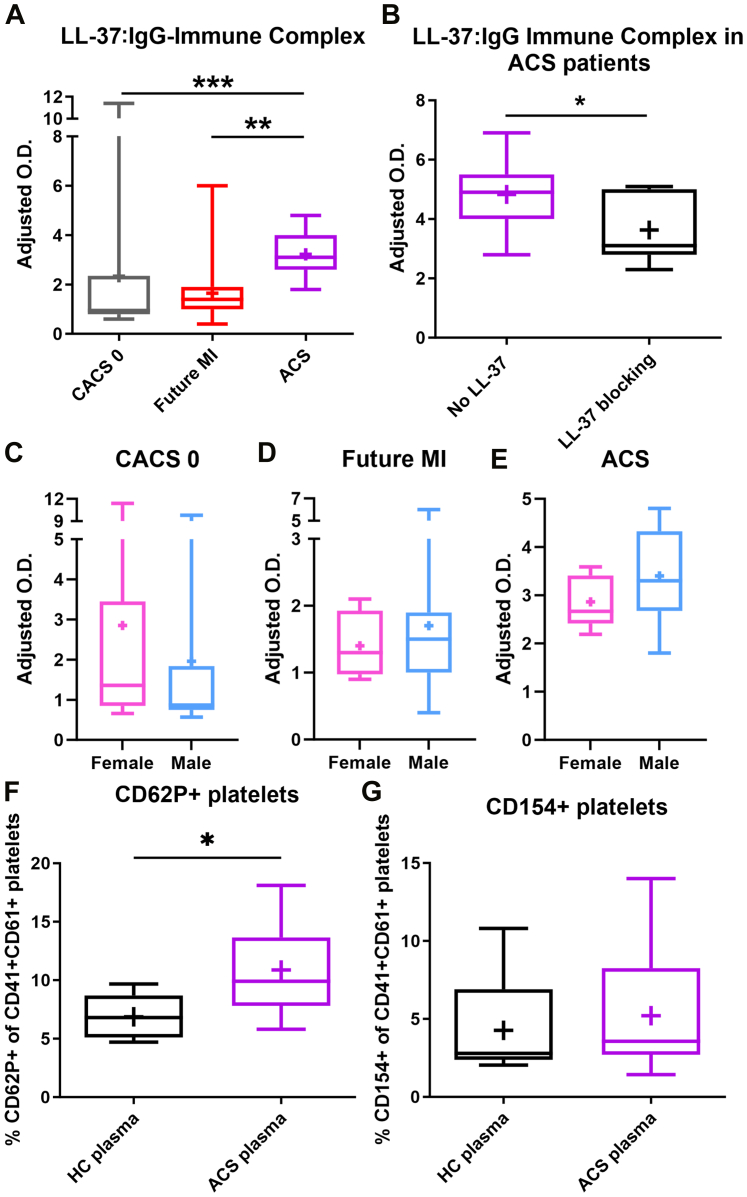
LL-37 IgG IC and Platelet Activation (A) LL-37 IgG-IC in subjects with zero (0) CACS (grey), Future MI patients (red), and ACS patients (purple). (B) Validation of LL-37 IgG-IC ELISA using LL-37 peptide blocking of anti–LL-37 capture antibody. N = 7 each. (C to E) LL-37 IgG-ICs compared by sex among the different patient groups. (F) CD62P+ platelets from healthy donors incubated with 5% plasma from pooled healthy control subjects (HC; black) or individual ACS patients (purple). HC, N = 5; ACS, N = 13. (G) CD154+ platelets from healthy donors incubated with 5% plasma from pooled HC subjects or individual ACS patients. Experiments were repeated at least 3 times with each data point an average of duplicates. (A) Kruskal-Wallis with Dunn test; (B) paired *t* test; (F) unpaired *t* test. ∗∗∗*P* < 0.001; ∗∗*P* < 0.01; ∗*P* < 0.05. ELISA = enzyme-linked immunosorbent assay; other abbreviations as in Figure 1.

There were significantly elevated LL-37 IgG-ICs in ACS patients compared with subjects with CACS = 0 and Future MI patients (Figure 2A). To validate the IgG-IC assay, LL-37 peptide was used to preblock the capture antibody. There was significant attenuation of the LL-37 IgG-IC signal in ACS plasma with LL-37 peptide preblocking (Figure 2B). The results suggest that although LL-37 IgG levels were less in ACS patients compared with Future MI, this is due in part to increased LL-37 IgG-IC formation. No differences were noted between female and male LL-37 IgG-IC levels in the different patient groups (Figure 2C to 2E).

### ACS plasma activates platelets from healthy donors

Platelet activation by immune complexes may be important in the pathophysiology of ACS. We evaluated the propensity of ACS plasma samples to promote platelet activation using platelets from healthy donors. There was significantly increased CD62P+ platelets after incubation with ACS plasma compared with pooled plasma from healthy subjects (Figure 2F). There was no difference in CD154+ platelets with ACS plasma compared with plasma from healthy subjects (Figure 2G). The results support the presence of soluble factors in ACS plasma that increases platelet activation marked by CD62P. To test the role of LL-37 IgG-ICs in ACS patient plasma in platelet activation, LL-37 IgG-ICs were immune-depleted from ACS plasma.

### Depletion of LL-37 IgG-ICs reduced ACS plasma activation of platelets

**Figure 3:**
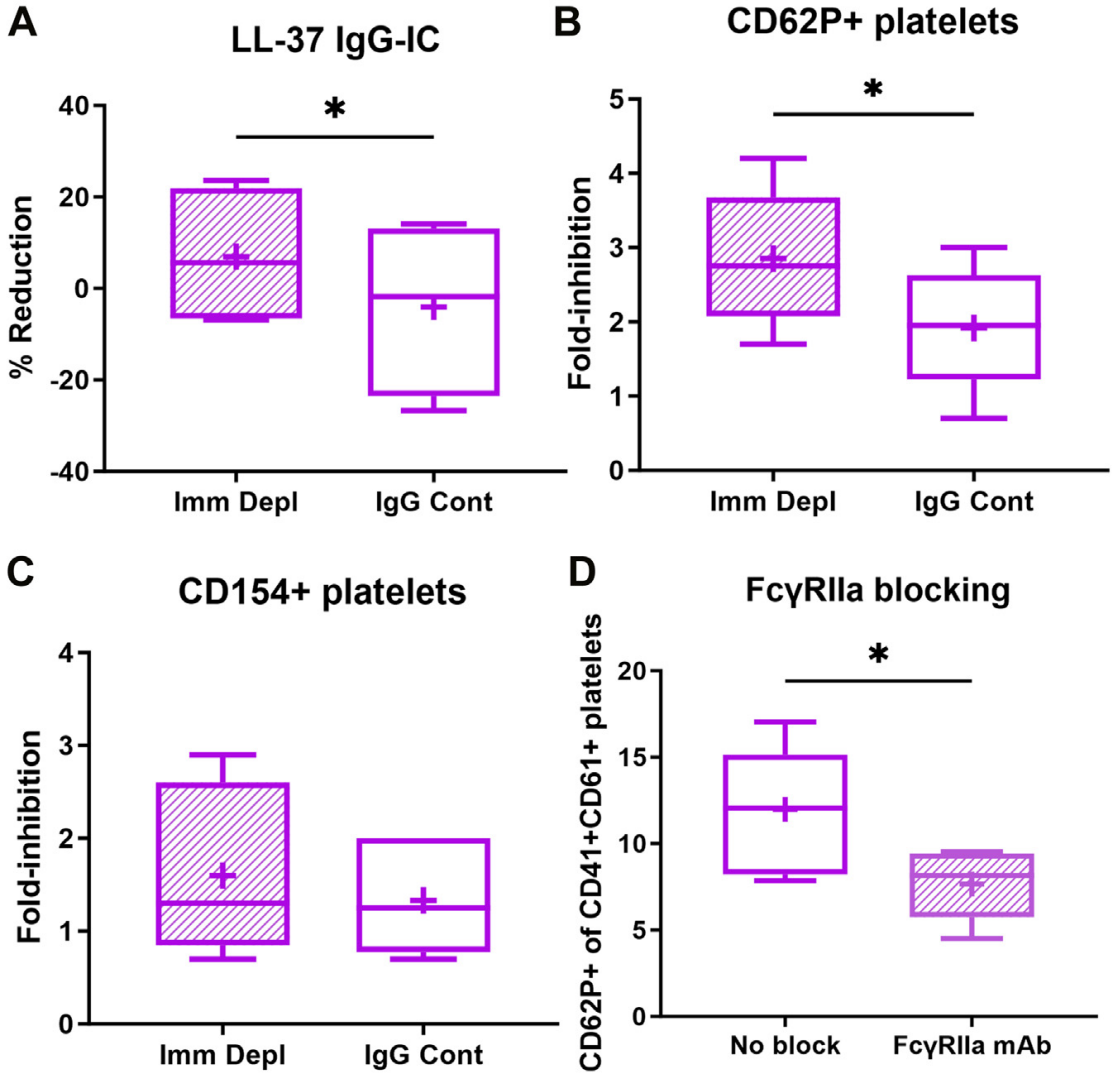
Immune Depletion of LL-37 IgG-IC and FCɣRIIa Blocking LL-37 IgG-ICs were immune depleted from ACS patient plasma using anti–LL-37 antibody conjugated to magnetic beads (Imm Depl) and the immune-depleted plasma used to stimulate healthy donor platelets. IgG-conjugated magnetic beads served as controls (IgG Cont). (A) Percent reduction in LL-37 IgG-ICs in immune-depleted patient plasma measured using ELISA (N = 4). Fold-inhibition of (B) CD62P+ platelets and (C) CD154+ platelets relative to no magnetic bead incubation (N = 6 each); ∗*P* < 0.05, paired *t* test. (D) Effect of FcɣRIIa monoclonal antibody blocking on CD62P+ healthy donor platelets stimulated with ACS plasma. N = 6 each; ∗*P* < 0.05, paired *t* test. FcɣRIIa = Fc gamma receptor 2a; other abbreviations as in Figures 1 and 2.

LL-37 IgG-ICs in ACS plasma were depleted using sheep anti–LL-37 antibody conjugated magnetic beads. Nonspecific IgG-ICs binding to the antibody host IgG was controlled for using sheep IgG conjugated to magnetic beads. Immune depletion resulted in a modest but significant reduction of ACS plasma LL-37 IgG-IC (Figure 3A). Importantly, immune depletion of ACS plasma LL-37 IgG-ICs had a significant 3-fold inhibition of CD62P+ platelets compared with 2-fold inhibition by control IgG (Figure 3B). There was no difference in CD154+ platelets by immune depletion of ACS plasma LL-37 IgG-ICs compared with IgG (Figure 3C).

### FcɣRIIa blocking reduced ACS plasma activation of platelets

FcɣRIIa expressed by platelets is one potential mechanism for LL-37 IgG-ICs to activate platelets. A mouse anti-human FcɣRIIa monoclonal antibody (monoclonal antibody clone IV.3) preincubated with healthy donor platelets was used to evaluate the role of FcɣRIIa in activation of healthy donor platelets by ACS plasma. Individual plasma from the 4 ACS patients with the highest LL-37 IgG-ICs were used to ensure healthy donor platelet activation. Preincubation with FcɣRIIa monoclonal antibody significantly reduced CD62P+ platelets (Figure 3D). The results support a role for FcɣRIIa in mediating LL-37 IgG-IC activation of platelets through increased CD62P and provide further evidence of the potentially detrimental role of LL-37 IgG-ICs in ACS.

## Discussion

The current study extends our previous report on the potential role of self-reactive immune response to the cathelicidin antimicrobial peptide LL-37 in ACS.^12^ The study demonstrates that: 1) LL-37 IgG is significantly elevated in intermediate-risk patients who have a future MI; 2) ACS patients have increased LL-37 IgG-ICs; and 3) LL-37 IgG-ICs in ACS patients activates platelets from healthy donors. The combined results support a detrimental role for the adaptive immune response to LL-37 in ACS.

The LL-37 IgG autoantibody levels in plasma samples of study subjects was not related to the extent of ASCVD but instead was significantly higher in patients who had a future MI, suggesting that high LL-37 IgG levels may increase risk for a future acute ASCVD event. Furthermore, we noted the reduced LL-37 IgG autoantibody levels in ACS patients, which was associated with increased detection of LL-37 IgG-ICs, suggesting that the formation of antigen-specific antibody-immune complexes may be functionally important in ACS. This is supported by our findings that LL-37 IgG-ICs promote the activation of platelets from healthy donors marked by increased CD62P+ platelets. Depletion of the LL-37 IgG-ICs resulted in reduced platelet activation. Our results further support a role for FcɣRIIa in platelet activation induced by ACS plasma demonstrated by the FcɣRIIa blocking experiment. FcɣRIIa avidly binds to IgG-ICs as compared with low affinity for monomeric IgG.^2^^,^^26^ Given that platelets are the most abundant carriers of FcɣRIIa in the blood, it is a key element in IgG-IC–induced platelet activation.^2^ The results suggest that LL-37 IgG form ICs with LL-37 in the acute stage and activate platelets mediated in part by FcɣRIIa, which potentiates thrombosis. Taken together, our report supports a potentially detrimental role of LL-37 IgG autoantibodies in ASCVD by increasing the risk for ACS. This is consistent with the report that FcɣRIIa binding to IgG-ICs increases platelet activation and promotes thrombosis in systemic lupus erythematosus.^15^ The role of LL-37 IgG-ICs specifically in adaptive immunothrombosis is yet to be reported but has been demonstrated to promote pathogenic bone resorption in RA.^27^

LL-37 is a component of NETs and itself promotes platelet activation and augments thrombus formation.^28^^,^^29^ Our report adds another layer to the detrimental role of LL-37 in thrombosis by defining a role in the immunothrombotic response in ACS mediated by LL-37 autoantibodies. The combined studies suggest that several properties of LL-37 conspire to bypass endogenous mechanisms of thrombus resolution^30^ and lead to the propagation of pathologic thrombosis in ACS.

Although the potentially detrimental role of adaptive immune responses to LL-37 in ASCVD demonstrated by our prior report^12^ and the current study is novel, LL-37 itself has been reported to bind low-density lipoprotein (LDL) and may promote ASCVD.^31^, ^32^, ^33^, ^34^ Importantly, the link between autoimmune/autoinflammatory diseases such as psoriasis and LL-37 was highlighted in a recent report by Nakamura et al^34^ demonstrating that LL-37 binding potentiates uptake of LDL by macrophages thereby altering the transcriptional response and promoting the formation of foam cells, adding further to the inflammatory role of LL-37 specifically in ASCVD. Structural modeling studies in their report suggest that LL-37 binding results in changes in LDL conformation increasing its size and potentially mediating its increased uptake by classic LDL receptors. Transgenic expression of LL-37 in apoE−/− mice increased aortic atherosclerosis further supporting its detrimental role in ASCVD.^34^

### Study Limitations

A limitation of the study is the small number of patients included in the analysis. These patients are from two different subcohorts selected based on the occurrence of MI during the follow-up period. In addition, comparison was made between patient samples collected from different studies. Consideration must be made for the possibility of selection bias and other covariates not balanced between the groups. The results will need to be validated with a future randomized trial or with a larger prospective cohort study with proper control of baseline imbalance. Another limitation is the lack of increase in CD154 of healthy donor platelets treated with ACS plasma. CD154 is often associated with platelet activation and thrombosis. It is possible that the in vitro assays we performed did not replicate the complex cascade that occurs in ACS patients, or that LL-37 IgG-ICs are more specific to prime CD62P signaling and that CD154 is activated by a different pathway that was not recapitulated in our experiments. Platelet CD62P is an important element in arterial thrombosis, promoting platelet-leukocyte aggregates,^35^ through rapid exposure of tissue factor to monocytes,^36^ and is associated with the extent of myocardial injury and rate of 30-day major adverse event in STEMI patients.^37^

## Conclusions

Our study demonstrates a novel pathway of platelet activation in ACS mediated by LL-37 IgG autoantibodies through the formation of ICs potentially promoting excessive inflammatory immunothrombosis. Neutrophil activation during immune and/or traumatic challenges, including coronary atherosclerotic plaque erosion or rupture, leads to NET expulsion of LL-37 autoantigens. The increased levels of LL-37 IgG predisposes the generation of LL-37 IgG-ICs that bind to platelets through FcɣRIIa, resulting in platelet activation and promotion of excessive thrombus formation.

Perspectives **COMPETENCY IN MEDICAL KNOWLEDGE:** The report defines a mechanism for platelet activation induced by an autoantibody response specific for a self-antigen in NETs that may contribute to thrombosis in ACS. Autoantibody levels may be clinically relevant in risk assessment of ASCVD patients.

**TRANSLATIONAL OUTLOOK:** Platelet activation and thrombosis are key elements of the attendant risk of ACS in ASCVD patients. Antiplatelet therapy can be enhanced by a clearer understanding of the factors involved in platelet activation and thrombosis mediated specifically by autoantigens in NETs and autoantibody-immune complexes.

## Funding Support and Author Disclosures

Funding was provided by The Heart Foundation, Eisner Foundation, Peterson Foundation, Corday Foundation, Spielberg Fund (Los Angeles, California; Dr Shah); The Eleanor and Harold Foonberg Endowed Chair in Cardiac Intensive Care Fund (Los Angeles, California; Dr Cercek); Academic Affairs Department, Cedars-Sinai Medical Center (Los Angeles, California); The Lydia Kitner Scholarship for Advanced Cardiovascular Training; the Milovicz grant for the Development of the Future Generation of Leaders in Medicine; and the Prof. Arieh Roth Scholarship from the Working Group in Acute Cardiac Care of the Israel Heart Society (Israel; Dr Chernomordik). The authors have reported that they have no relationships relevant to the contents of this paper to disclose.
